# Exploration of Factors Associated with Reported Medication Administration Errors in North Carolina Public School Districts

**DOI:** 10.1177/10598405221127453

**Published:** 2022-09-21

**Authors:** Nakia C. Best, Ann O. Nichols, Bosny Pierre-Louis, Jessica Hernandez

**Affiliations:** 1 Sue & Bill Gross School of Nursing, The 8788 University of California, Irvine, Irvine, CA, USA; 2 Retd., Division of Public Health, Children & Youth Branch, NC Department of Health & Human Services, Raleigh, NC, USA; 3 Novion Analytics, Durham, NC, USA

**Keywords:** school health, school nurse, medication administration, medication management

## Abstract

School nurses are pivotal to the safety of school-aged children, particularly those who receive medications in the school setting. The purpose of this study was to explore factors associated with medication administration errors in North Carolina school districts between 2012/2013 and 2017/2018. A longitudinal study using repeated measures analysis of school health services data collected in the North Carolina Annual School Health Services and Programs Survey was conducted. Over time, the number of medication errors (*p* = .001) and number of medication corrective action plans (*p* < .0001) trended upwards. There was also an increase in medication errors when the number of schools in a district was higher (*p* < .0001). Conversely, there was a decrease in corrective action plans when school nurses were directly employed by the school district (*p* = .0471). We implore school disticts to consider the important role of school nurses to keep kids safe, healthy, and ready to learn.

More than 40% of school-aged children and adolescents in the United States live with at least one chronic health condition diagnosis, such as asthma, diabetes, and attention deficit hyperactivity disorder (Centers for Disease Control and Prevention, 2021). These conditions often include complex medication regimens that require medication administration during the school day. During the school year students also may receive medications for acute illnesses, emergencies, or on an as needed basis (prn), while attending school. Ensuring optimal management of student medication regimens is vital to health and safety while improving the ability to learn and play, and overall quality of life (Lowe et al., 2022).

As a non-healthcare-oriented institution, the management of medications in the school setting is complex. School nurses provide medication administration training and education for students, parents, and school employees on topics such as administration of oral and topical (prescription, nonprescription, emergency) medications, student medication self-administration (self-carry), medication storage and security, documentation, and medication error prevention (Little et al., 2018) and remediation. School nurses work to maintain safe medication practices while often using multiple levels of policies, procedures, and protocols. Responses from 6,127 school nurses in a national study on medications in schools indicated that 12.3% worked in locations with school-specific policies, 84.8% with school district-level policies, and 43.5% with state-level policies (Maughan et al., 2018). Adding to the challenges to be overcome, school nurses must ensure that school-specific and school district policies align with state and federal laws and regulations (National Association of School Nurses [NASN], 2021).

Without adequate safeguards and oversight in place, management of medications in the school setting introduces risk for students and possible liability for schools. Medication errors are preventable events that may cause harm to students or lead to inappropriate medication use (National Coordinating Council for Medication Error Reporting and Prevention, 2007). Maughan et al. (2018) found that 15.3% of school nurses surveyed reported that a medication error had occurred during the school year. They also found that the most common medication error was a missed dose, like previous studies (Clay et al., 2008; Ficca & Welk, 2006; Maughan et al., 2018; McCarthy et al., 2000)

Generally, medication administration and oversight have been the responsibility of the school nurse while the student is attending school. However, only 39% of schools in the U.S. employ full-time school nurses, 35.3% of schools employ part-time school nurses, and 25.2% of schools do not employ a school nurse at all (Willgerodt et al., 2018). In North Carolina, although all public schools in Local Administrative Agencies (LEAs; i.e., school districts) have access to a school nurse, only 12% of school districts provide the services of one full-time nurse in every school (North Carolina Division of Public Health, 2020). When there is no school nurse available, it is common for school staff without a healthcare license (unlicensed assistive personnel [UAP]) to administer medications under the delegation of the school nurse (Lowe et al., 2022). Examples of UAPs who are trained to administer medications to children in schools include secretaries, health aides, teachers, and principals (Lowe et al., 2022). During the 2015–2016 school year, 76% of the medications given to students in North Carolina were administered by UAPs (North Carolina General Assembly/Program Evaluation Division, 2017).

This is concerning since medication errors are more likely to be made by UAP than by school nurses (Maughan et al., 2018; McCarthy et al., 2000).

Although the evidence suggests that medication administration accuracy needs to be improved, research explaining factors that affect the incidence of medication errors is still needed. In fact, The National Association of School Nurses 2021–2022 research priorities include analysis of existing data that examine structural measures on student outcomes, such as medication errors. The purpose of this study was to explore factors associated with reported medication administration errors in North Carolina (NC) school districts between 2012/2013 and 2017/2018.

## Framework

**Figure 1.**
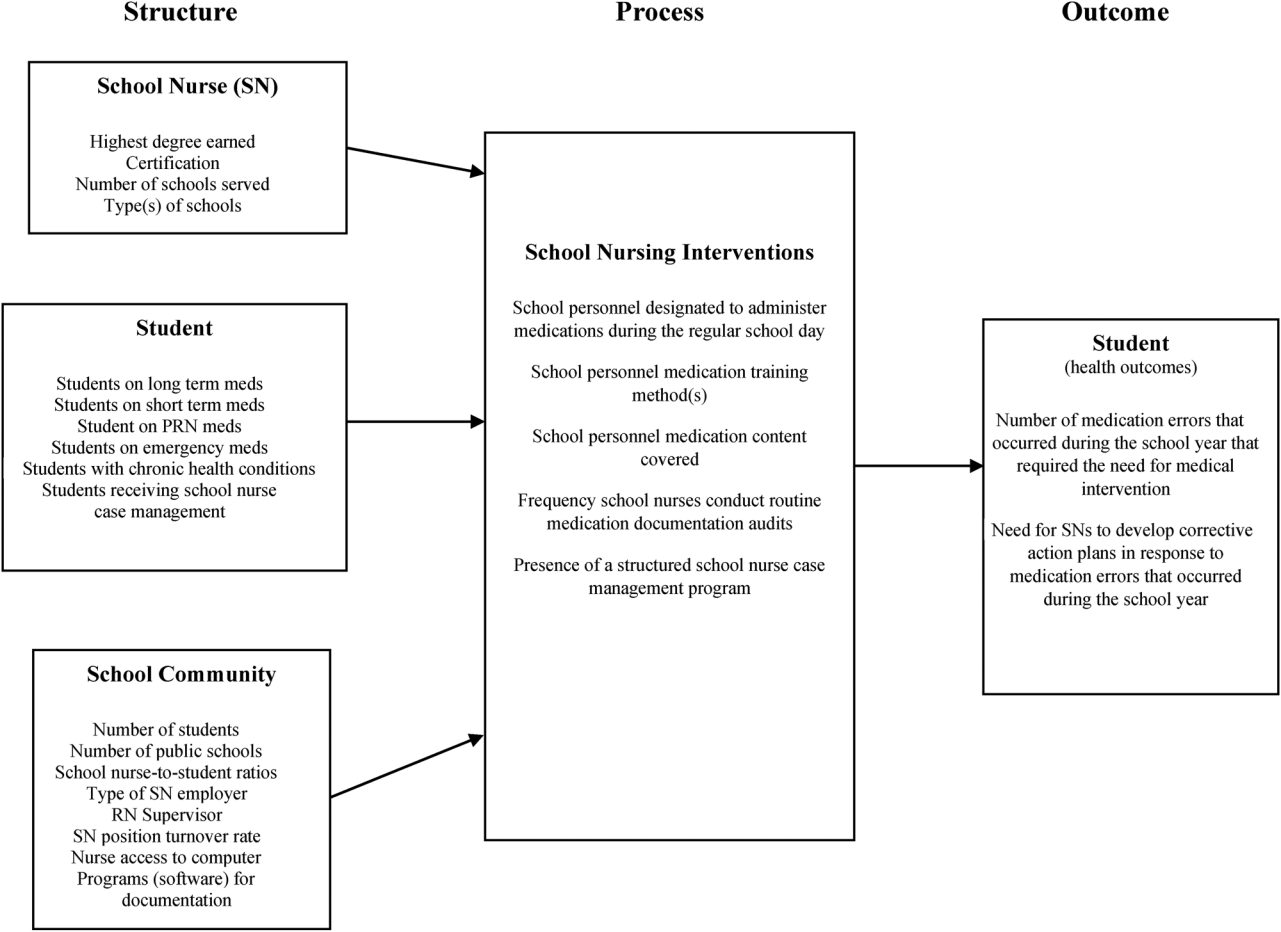
3S model for medication administration.

This study was guided by the 3S (Student-School Nurse-School Community) Model (Wolfe et al., 2019), developed to provide a visual representation of how to categorize and identify school health data. The 3S Model is an adaptation of Donabedian's structure, process, and outcome model. The 3S Model was used to categorize school nurse, student, and school community data (structure), school nurse intervention data points that represent work school nurses conducted to maintain/improve student health (process), and student data points that demonstrate changes that have occurred over time (outcome; Figure 1).

## Methods

To address NASN research priorities, we used a longitudinal study design to conduct a repeated measures analysis of school health services data collected in the North Carolina Annual School Health Services and Programs Survey. This survey represents aggregated district level data that are collected and reported by school nurses each school year. The Annual Survey was developed and managed by data specialists and the School Health Nurse Consultant Team in the Children and Youth Branch of the North Carolina Division of Public Health. The team consists of the State School Health Nurse Consultant and six Regional School Health Nurse Consultants, directly managed by the state position. The Consultant Team provides consultation and technical support to school nurses and other school staff to ensure that student health, wellness and needs are addressed for optimal educational access. The survey data collection process provides instructions with standardized definitions that assist with collecting and reporting the data accurately. Among the data collected are a variety of school nurse and school community characteristics, health conditions of students served, and health services provided. School nurses representing all 115 North Carolina public school LEAs reported data each school year. While these data are representative of students enrolled in public elementary, middle, and high schools who received nursing services, other student specific data, such as demographic data, are not collected. Although it has changed over time, the North Carolina Annual School Health Services Report survey has existed since its inception in 1996. This study was determined to be exempt by the University of California, Irvine Institutional Review Board (IRB# 14097).

### Sample

School health services data for this study covered school years 2012/2013 through 2017/2018. The sample for this study included all North Carolina school LEAs (*N* = 115). With development of the survey process over this period some data were not available for all years including data on medication errors, which was added to the survey in school year 2012–2013.

### Procedures

Data for each school year were cleaned and aggregated into one dataset to represent all 115 school districts. The dataset was examined for any missing items and follow-up was conducted to determine if those data were available. Upon cleaning the data, the first author discovered school year 2012/2013 data were missing. Updates were made to the dataset when the 2012/2013 data were provided by the second author. Some of the individual school nurse characteristics were missing and could not be located, however, the statistician accounted for this in the statistical analysis.

### Variables

School nurse was defined as a registered nurse who served the general school population full-time or part-time and did not work solely as an administrator. Students were defined as school-aged children and adolescents enrolled in public elementary (K-5th grades), middle (6th-8th grades), and high (9th–12th grades) schools. Average school nurse/student ratios were based on full-time equivalents (FTE positions budgeted for school nurses) that worked in the school districts. Time was defined as school years 2012/2013 to 2017/2018. Data of interest from all 115 school districts were used and are reported in Table 1.

**Table 1.** Selected Characteristics of North Carolina School Districts by School Year.

	2012	2013	2014	2015	2016	2017
School nurses, *n*	1,290	1,292	1,353	1,357	1,368	1,379
Number of schools served						
1 school	446	478	580	551	582	568
2 schools	447	441	416	468	507	562
3 or more schools	318	328	314	282	273	242
Highest degree						
Associate degree	135	128	129	124	129	113
Baccalaureate degree	985	1,017	1,070	1,076	1,070	1,121
Master's degree	92	89	90	90	83	90
Other degree	23	22	17	13	12	10
Percent nationally certified	49.8%	51.1%	50.9%	51.4%	52.9%	54.2%
Students, *n*						
Long term medications	23,048	24,205	57,926	24,118	29,937	29,511
Short term medications	6,559	6,335	44,561	5,918	5,092	5,104
Non-emergency medications	32,693	28,706	35,642	32,031	31,596	32,569
Emergency medications	63,663	67,271	67,563	72,176	69,209	85,059
Students with chronic/complex conditions	274,187	221,437	244,351	213,069	258,692	245,807
Students that received case management for chronic/complex health conditions	6,102	5,090	6,450	7,619	6,836	6,553
School community (*n* = 115)						
Total number of students	1,427,281	1,434,180	1,433,592	1,459,852	1,428,051	1,422,305
Total number of public schools	2,427	2,441	2,443	2,446	2,447	2,447
Full time equivalents	1,212	1,236	1,288	1,318	1.332	1,348
Average Nurse/student ratio	1,177	1,160	1,112	1,086	1,073	1,055
Districts that provide RN supervisor (%)	41 (35.7)	42 (36.5)	43 (36.5)	42 (36.5)	40 (34.8)	38 (33)
Districts that directly employ nurses (%)	84 (73)	85 (73.9)	85 (73.9)	86 (74.8)	86 (74.8)	86 (74.8)

*Note.* For school nurse characteristics, there were some missing data

#### Structure

*School nurse* variables included: highest degree earned, certification (does the school nurse have certification from the National Board for Certification of School Nurses (NBCSN) [yes/no], number of schools served by each school nurse, and type of school (elementary, middle, high, combination). *Student* variables included: number of students on long term, short term, PRN (non-emergency), and emergency medications. We also included the number of students with chronic health conditions and of those, the number who received school nurse case management (structured program for students with chronic/complex health conditions requiring regular school nurse contact with the student and their family, teachers, and/or health care providers; Best et al., 2021a). *School community* variables included: total number of students, number of public schools within the district, average school nurse/student ratios (based on full-time equivalents budgeted for school nurses) employed in the school districts, type of employing agency of school nurses (school district, health department, hospital/combination), registered nurse (RN) supervisor (is the supervisor of school nurses a RN [yes/no]), school nurse turnover rate (percentage of school nurse positions that were vacated during the school year), nurse access to computer at work for daily work (e.g., Internet and email) and nursing documentation (yes/no), and software programs used for documentation.

#### Process

**Figure 2.**
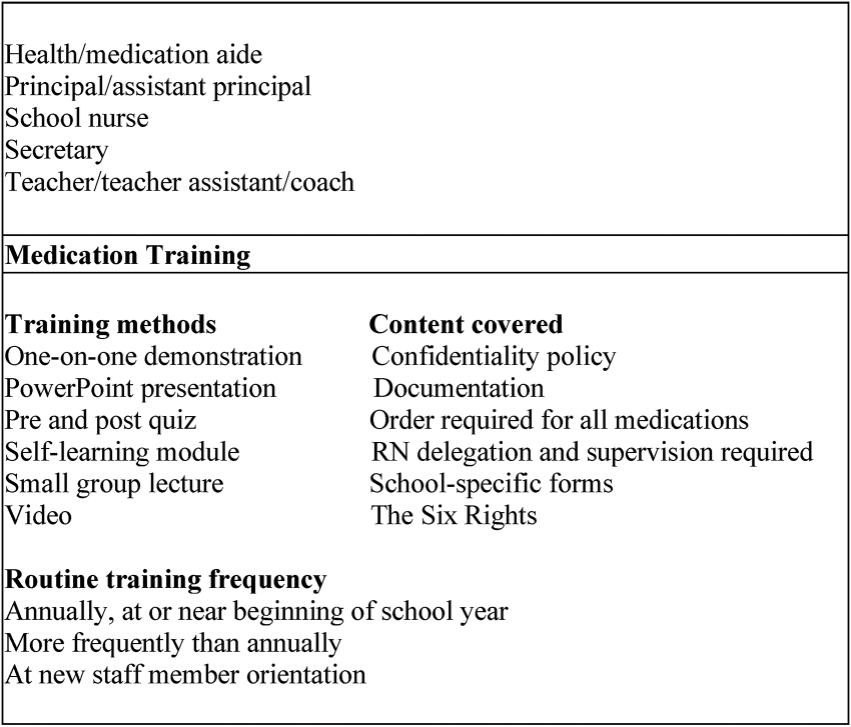
Process variables.

Process variables included school nurse medication administration-related interventions: school personnel designated to administer medications, medication training (methods, content, frequency), frequency of medication documentation audits school nurses conducted, and presence of a structured school nurse case management program (Figure 2).

#### Outcome

Outcome variables were the number of medication errors that required medical intervention and the need to develop corrective action plans (CAP) in response to medication errors discovered during audits at any time of the year (yes/no). In North Carolina school health program reports, a corrective action plan was defined as a plan designed to provide one or more corrective actions in response to a medication variance discovered at any time during the school year that violated any of the six rights of medication administration. An example of a medication error is the administration of a medication to the wrong student. Corrective action for the error in this example could be improving methods of student identification prior to medication administration and training staff on those methods. School nurses write CAPs based on their assessment of the breakdown in medication administration that resulted in the error to ensure correction for future administration; the plans may include repeat training and assuring competence of the person administering medications.

### Data Analysis

The unit of analysis was the school district (LEA) level. Each model included time (entered as a categorical variable) as a fixed effect. Descriptive statistics were computed for health services and programs and health outcomes at each school year and overall. Longitudinal graphical displays (mean profiles, histograms of the counts) were created to identify patterns in the health services, programs, and outcomes data over time, and to inform subsequent modeling decisions. All tests of hypotheses were two-tailed, and a *p*-value ≤.05 was considered statistically significant. No adjustment was made for multiplicity. All analyses were performed using SAS® software (Version 9.4 or higher; SAS Institute, Inc., Cary, NC, USA).

Medication errors were operationally defined with two variables, the first being the number of medication errors in each school district. To model this non-negative discrete count outcome variable, we adopted the generalized linear model framework by way of generalized estimating equations (GEE) with Poisson distribution and the log link function (Diggle et al., 2002; Liang & Zeger, 1986). A multi-step process was adopted. First, school nurse, student, school community, and school nurse interventions variables and their effect on the number of medication errors were analyzed separately. Each model included fixed terms for the specific factor characteristic of interest and time. In a subsequent step, variables with a *p*-value of.10 from each model were simultaneously entered as covariates in a multivariate longitudinal Poisson GEE model. In all GEE models, a log link and a Poisson distribution were used, and an exchangeable correlation structure was assumed. Evaluation of the Poisson models for possible occurrence of overdispersion was conducted, and a scaling factor was applied to the model covariance matrix to correct for any detected overdispersion.

The second variable to operationalize medication errors was the need for school nurses in the school district to develop corrective action plans in response to medication errors during the school year. This binary outcome (yes/no) was analyzed via a generalized linear model framework by way of GEE with a logistic distribution and log link function. The fixed effects for the logistic GEE models and the testing strategy proceeded in the same manner as for the Poisson GEE models specified above. The outcome was modeled as the probability = 1 of a need in the LEA to develop one or more corrective action plans in response to a medication error discovered at any time in the year.

No imputation was carried out for missing data. Variables with excessive missing values were not entered into the models. Some categorical variables were removed from the analysis because they took on a single value (usually 1), which led to quasi or complete separation of data points making the regression models unstable. Finally, a few other variables were dropped out of the models because of collinearity issues with remaining variables.

## Results

### Structure

#### School Nurses

The study sample included all 115 North Carolina school districts. Between 2012/2013 and 2017/2018, the percentage of school nurses with a Baccalaureate degree increased from 76% (985 out of 1,290 nurses) to 81% (1,121 out of 1,379 nurses). The number of school nurses who held national school nurse certification increased from 643 (49.8%) to 748 (54.2%). Each year, most school nurses served one to two schools. Lastly, nurses who served three or more schools decreased from 25% to 18% (see characteristics in Table 1).

#### Students

On average each school year, 31,458 students received long-term medications (more than 3 weeks) and 12,262 received short-term medications (less than 3 weeks). On average, each school year 32,569 students had provider orders for PRN (non-emergency) medications, and 70,824 students had provider orders for emergency medications. On average, there were 242,924 students with chronic and/or complex health care needs receiving services each school year. Of those, 6,441 students with chronic and/or complex health care needs received structured case management from school nurses each year.

#### School Community

By 2018, 1,422,305 million students attended one of North Carolina's 2,447 LEA public schools. Between 2012 and 2018, the number of school nurse FTEs increased from 1,212 to 1,348 and the average school nurse-to-student ratio decreased from 1:1,177 to 1:1,055. Over time, the number of school districts that employed school nurses (versus employment through a health department or hospital) increased from 84 (73%) in 2012 to 86 (74.8%) in 2018. On average, 41 (36%) of the school districts utilized a registered nurse to supervise the school district's nurses. On average, 40 (35%) school districts had 1 to 2 empty school nurse positions, annually. All 115 school districts provided nurses with access to a computer at work for daily work. Between 2012 and 2018, the number of school districts where nurses used a computer for nursing documentation increased from 51 (44%) to 83 (72%). In 2012, most school districts did not have a standardized program or platform for nursing documentation, however by 2018, 72 (63%) school districts reported use of electronic health records (e.g., Health Master, Health Office, Patagonia, CareDox) and PowerSchool.

### Process (School Nursing Interventions)

Annually, the most common personnel designated to administer medications during the regular school day were secretaries (112 [97%] school districts) and teachers (107 [93%] school districts). The most common medication training methods for school personnel were one-to-one demonstration (107 [93%] school districts) and small group lecture (88 school districts), while self- training (33 [29%] school districts) was used the least. By 2018, all school districts” medication training content included documentation, school specific forms, The Six Rights, and the requirement of a provider order for all prescription and non-prescription medications. Annual medication training at or near the beginning of the school year was the most common frequency for required medication training (100 [87%] school districts). Lastly, school nurses most often conducted routine medication documentation audits quarterly (71 [62%] school districts) or monthly (21 [18%] school districts) in LEAs that reported an audit process in place.

### Outcome

#### Medication Errors

As noted in Table 2, across all 115 school districts an average of 78 medication errors that required medical intervention were reported as occurring annually. On average, about 31 (27%) school districts reported developing one or more corrective action plans in response to a medication error discovered during each school year that rose to the level of requiring that intervention. We found an inverse relationship between audit frequency and number of medication errors. More specifically, the rate of medication errors decreases by a factor of exp (−.3428) = .71 as audits become more frequent. These results, however, did not reach statistical significance at the alpha = 0.05 level.

**Table 2.** Reported Medication Errors and Corrective Action Plans.

	Medication Errors	Corrective Action Plans
School year		
2012–2013	49	24
2013–2014	37	22
2014–2015	39	22
2015–2016	160	34
2016–2017	52	34
2017–2018	128	49

### Associations with Medication Errors

#### Number of Medication Errors

In Table 3 variables for school nurse, student, and school community characteristics, and nurse medication-related interventions were examined separately and jointly for their associations with number of medication errors. In the model that included student characteristics (ratio, total number of students, number of students who received services for a chronic condition, and number of students who take long term, prn, and emergency medications) as lone exploratory variables, the number of medication errors trended towards an increase over time (*p* = .0391). In a second model that included school community characteristics (ratio, RN supervisor, turnover rate, number of schools in the district, computer availability, type of supervising agency) as predictors, when there was a higher number of schools in the district, there was an increased rate of medication errors (*p* = .0274). Conversely, when school nurses were directly employed by the school district, there was a decrease in the rate of medication errors (*p* = .0269). In the model that included nurse medication-related interventions (designated staff trained to administer medications, training and content methods, training frequency, school nurse case management program) the rate of medication errors increased when the medication training method was self-training (*p* = .041) or PowerPoint presentation (*p* = .0007), and when medication training was completed during orientation (*p* = .0135). Finally, in a multivariate model simultaneously adjusting for student, nurse medication-related interventions, and school community characteristics, time and number of schools located in the district continued to have an impact. Controlling for all other variables in the model, the rate of reported medication errors increased slightly over time (*p* < .0001), furthermore, when the number of schools in the district was higher, the estimated rate of medication errors was higher (*p* < .0001).

**Table 3.** Multivariate Models of Outcome Measures, 2012–2018.

Model	Variables	Estimate	95% CI	*p*-Value
Associations with Number of Medication Errors	School year	0.0018	0.0009, 0.0027	.001
	Number of schools in school district	0.0070	0.0042, 0.0098	<.0001
Associations with Number of Medication Corrective Action Plans	School year	0.0033	0.0019, 0.0046	<.0001
	Training more frequently than annual	0.6054	0.1010, 1.1099	.0187
	School nurse employed by school district	−0.5802	−1.1529, −0.0074	.0471

Note. CI = Confidence interval.

#### Development of Medication Corrective Action Plans

Using the same modeling strategy as the one adopted for assessing the impact of the number of medication errors, separate models were built to examine the effect of student, nurse medication-related interventions, and school community data on the reported development of one or more corrective action plans in response to a medication error during the school year. It would be expected that reported corrective action plans closely represent their need in addressing medication errors since use of these plans is a standard in North Carolina Schools (NCDPH, 2021). Over time, in all models there were more districts who developed corrective action plans (*p* < .0001). In the model built for student characteristics, on average, the longer students were on a medication, the less likely corrective action was needed (*p* = .0031). In the model that included nurse medication-related interventions, the factors associated with the need to develop a corrective action plan were medication training conducted annually (*p* = .0338), training more frequently than annually (*p* = .0021), and the presence of structured school nurse case management for chronic health conditions (*p* = .0361). In the model that incorporated only school community characteristics, when there was a higher number of schools in the district, there was a higher need to develop a corrective action plan (*p *= .0274). Also, when school nurses were directly employed by the school district, there was a decreased need to develop a corrective action (*p* = .0269). In the multivariate model that included all three sets of characteristics, the finding was that training provided more frequently than annually yielded a higher need to develop a corrective action (*p* = .0187). Conversely, when school nurses were directly employed by the school district, there was a decreased need to develop a corrective action (*p* = .0471).

## Discussion

The purpose of this study was to explore associations between medication administration outcome indicators (number of errors, development of corrective action plan) and school nurse, student, and school community characteristics and school nurse interventions among North Carolina school districts during school years 2012/2013 through 2017/2018.

The North Carolina State Board of Education requires national certification in school nursing within three years of initial hire as the credential for the professional school nurse (North Carolina Division of Public Health/School Health Unit, 2021). Enforcement of this requirement is the responsibility of district leadership. Although North Carolina school nurses showed modest growth in the percent of school nurses certified over the term of the study, this largely remained stagnant due to position turnover, the addition of new positions within three years of hire, and inconsistent local accountability. School nurses who have not achieved certification are often Associate Degree in Nursing (ADN) graduates who must complete a Bachelor's Degree in Nursing to meet certification eligibility criteria. School nurse certification may impact medication administration programs in North Carolina schools since achievement of specialty certification is associated with patient safety (Kendall-Gallagher & Blegen, 2009).

The major improvement for North Carolina school nurses noted was in the number of schools assigned due to decline in nurse to student ratio. Consistent growth occurred in the percent of school nurses serving fewer schools particularly in those serving three or more schools, due to a slow increase in school nurse FTEs across the state.

The rate of medication errors reported over time and the decision to develop a Corrective Action Plan (CAP) trended upwards in certain situations in the study. These included increasing numbers in the student characteristics model (ratio, enrollment, etc.) and the community characteristic of a higher number of schools in the district for nurses to cover. Higher number of schools was a persistent finding associated with increased error rates even when other variables were controlled. Similarly, Maughan et al. (2018) found medication administration errors were associated with school nurses responsible for more schools, while Ficca & Welk (2006) found medication errors were associated with school nurses who were responsible for more than three school sites. When a school nurse is assigned to multiple schools that assignment not only severely limits the time available to adequately oversee a program, but also presents other challenges that impact medication administration. In addition to working with different school staff dynamics and configurations, there can generally be wide variation in the culture and leadership support within each assigned school. Therefore, in being responsible for oversight of the medication administration program and training the school nurse must approach each school's program as a unique situation with its own individual strengths and challenges.

The impact of multiple school assignments and higher medication error rates may also be supported in the findings related to manner and timing of training for non-licensed staff in medication administration. When assigned to multiple schools it is not uncommon for North Carolina school nurses to use self-directed formats for needed training, particularly when new employees are hired and completing orientation requirements outside of the normal repeated training times for school staff. While alleviating the need to regularly dedicate time to this activity the association with increased rates of error may indicate the desirability for training methods that include higher level of involvement with the school nurse, particularly for new employees without previous experience from which to draw. Canham et al. (2007) support refresher instruction to meet identified needs of unlicensed assistive personnel (UAP) in addition to annual training. NASNs (2021) *Medication Administration Clinical Practice Guidelines in the School* provides UAP training recommendations including initial, annual, and ongoing timing for training and competency assessment, return demonstration of medication administration, and passing a written medication administration test.

Of interest is the finding of a decrease in the rate of medication errors and the development of corrective action plans when school nurses were directly employed by the school districts, as opposed to contracted for services through the local health department, hospital, or health alliance. In North Carolina there are often local differences in the level of integration of the school nurse into the school community and culture based on employing agency. School nurses that are contract employees often experience variable access to school data systems, inclusion in school staff expectations and meetings, participation in student related planning such as Exceptional Children and student support and are often viewed somewhat as outsiders. This difference in the degree of seeming a “part of the team” may in some way influence these and other factors related to services provided that would need further exploration. School nurses are vital school team members and integral to improving child health and education outcomes (Best et al., 2021b). Regardless of who they are employed by, they must be recognized for their expertise in identifying and intervening on issues that affect students, staff, and the school community (Allison et al., 2019).

Increase in corrective action plans over time is not a surprising finding in North Carolina and is likely a reflection of the increase in discovered medication errors that required intervention. Medication errors are often determined during audits, with audits being completed by school nurses. As the assignments of school nurses improves, that ability to attend to more detail with more time may well bring improvements in both completion and frequency of audits (Canham et al., 2007). It may not be that more errors are occurring that require intervention, but that having more nurses allows them to conduct audits more frequently and thoroughly in accordance with practice expectations and clinical guidelines (NASN, 2021).

As mentioned earlier, in the multivariate model that included all three sets of characteristics, training that was provided more frequently than annual yielded a higher need to develop a corrective action plan (*p* = .0187). While this may appear surprising, North Carolina school nurses provide additional training opportunities when errors are discovered, particularly during audits. Those additional training events have resulted in selection of “more frequent than annual” on district reports, even when those training events were not planned as part of the routine training program but were the result of discovered errors. This finding indicates a need for improved directions on the annual report document since districts may routinely plan to train annually in the absence of errors.

Students with chronic health conditions that received school nurse case management services represented an average 2.7% of all students with chronic health conditions. Case management services in North Carolina involve intense student engagement with the school nurse which includes assurance of optimal medication use and administration (Best et al., 2021a, 2021b). It is at times when school nurses are available and engaged in care that errors requiring a CAP appear to increase in the data—when providing medication training directly and when providing case management services. In the absence of the school nurse, medications are largely delivered by unlicensed school staff and some errors may go unidentified unless an audit is performed or a student's care is reviewed for some other reason, such as case management services. These findings further emphasize the importance of school nurse caseloads that allow the provision of school nursing services in a manner that allows direct service and is more consistent with safety and health (Daughtry & Engelke, 2018).

### Strengths and Limitations

This research study was designed in response to the National Association of School Nursing Research Request for Proposals (2019) from research projects to analyze existing school health services data. The North Carolina Annual School Health Services Report survey is a large, accreting, and very rich data set representing 1.5 million public school students in one of the largest systems in the United States. Strengths of the study included access to this data which enjoys an institutionally ingrained process for collection in North Carolina LEAs with 100% participation. As a result, the data represents responses from all LEA employed school nurses in North Carolina for the noted period.

Challenges that introduced limitations in the study design and required research team decision making included those that are often at issue when data is collected using different methodologies over time and for purposes that are primarily practice related and not intended for research. Those included possible data quality issues in assuring standardization over time and in the people involved in collection. In addition, over the school years included, the data collection process went from paper submission to a computer submission format, resulting in data transcription and storage in varying platforms with errors that needed to be reconciled prior to use. Data accuracy was an issue at times since lack of collection in real-time resulted in some fragmented data that impacted use, sometimes with no way to retrieve missing data. In addition, since there is no requirement to report medication errors in the state data, or to develop a Corrective Action Plan, the data likely under-represented the numbers of errors and plans that occurred during the study years.

### Implications for School Nursing Practice and Research

This is the first statewide longitudinal study of the structures and processes associated with medication administration outcomes in the North Carolina public school setting. School nurses in other states can use this information to model their school nurse, student, and school community structures, school nurse interventions (processes), and how they are associated with medication administration errors (outcomes).

Education is an integral part of school nursing practice (NASN, 2020). To ensure student safety, school nurses provide training and oversight to school staff who are delegated to administer medications in the school setting. We recommend school nurses and schools review training requirements, particularly the formats used for education (e.g., hands-on, return demonstration) and frequency of the training (e.g., initial, annual, ongoing assessment).

Daily access to a school nurse is particularly important for students who need to receive medications in school. It is generally accepted that when nurses serve fewer schools, they can provide more complete services and to maintain better oversight and support in those schools. The results of this study indicate that when there was a higher number of schools in a district for nurses to cover, it was associated with an increased rate of medication errors. School administrators and local policymakers must consider the safety of children in the school setting and how optimal school nurse workloads impact the health of children.

Researchers have acknowledged the need for national-level guidelines to address medication administration in the school setting (Kelly et al., 2003; Little et al., 2018; Lowe et al., 2022; Maughan et al., 2018). During the conduct of this study, NASN developed the *Medication Administration Clinical Practice Guidelines in the School* and *Medication Administration Implementation Toolkit (see references for access information)*. These efforts are monumental and offer an important opportunity for school nurses to use this decision-making tool to implement the recommendations in their schools and advocate for their local school districts and state policymakers to align medication administration policies and procedures with these evidence-based practice national guidelines. Potential next steps for this type of research would be to evaluate strategies used to increase adoption of NASNs medication administration guidelines, and how the implementation of the new guidelines is associated with decreasing the rate of medication administration errors.

## Conclusion

Our findings are consistent with the notion that the recommendation of one full-time professional school nurse in every school is pivotal to the safety of school-aged children, particularly those who receive medications in the school setting. We acknowledge the challenges and budgetary constraints school districts face; however, we implore them to consider the important role school nurses have in keeping kids safe, healthy, and ready to learn.
